# Effect of *Taraxacum officinale* extract on PI3K/Akt pathway in DMBA-induced breast cancer in albino rats

**DOI:** 10.1042/BSR20180334

**Published:** 2018-12-21

**Authors:** Mohamed Abdo Nassan, Mohamed Mohamed Soliman, Shimaa Ahmed Ismail, Samir El-Shazly

**Affiliations:** 1 Medical Laboratory Department, Faculty of Applied Medical Sciences, Turabah, Taif University, Saudi Arabia; 2 Department of Pathology, Faculty of Veterinary Medicine, Zagazig University, Egypt; 3 Department of Biochemistry, Faculty of Veterinary Medicine, Benha University, Egypt; 4 Department of Clinical Pathology, Faculty of Veterinary Medicine, Zagazig University, Egypt; 5 Department of Biotechnology, Faculty of Science, Taif University, Saudi Arabia; 6 Department of Biochemistry, Faculty of Veterinary Medicine, Kaferelsheikh University, Egypt

**Keywords:** Breast cancer, DMBA, Gene expression, Taraxacum officinale

## Abstract

Background: Breast cancer is one of the most prevalent types of cancer and a leading cause of death in women. Materials and methods: An experimental model of breast cancer was induced in female albino rats using single intragastric dose of 7, 12 dimethylbenz (α) anthracene (DMBA) in sesame oil (50 mg/kg b.wt). Four months after DMBA administration, incidence of breast cancer was confirmed by measuring cancer antigen 15-3 (CA15-3) serum levels. *Taraxacum officinale ssp. officinale* root extract (TOE) was administered in a dose of 500 mg/kg by oral gavage for 4 weeks after breast cancer incidence. Level of CA15-3 as one of the best known breast tumor markers was elevated in all positive breast cancer rats. The genetic effects of TOE on *Pdk1–Akt1–Pik3r1–Map3k1–Erbb2–PIk3ca* using semi-quantitative RT-PCR analysis were evaluated. In parallel, histopathological changes and immunohistochemical expression of Bcl2 in mammary gland tissues were examined. Results: Level of CA15-3 was normalized in DMBA group administered TOE for 4 weeks. Administration of DMBA increased expression of *Pdk1, Akt1, Pik3r1, Map3k1, Erbb2 and PIk3ca*. Treatment with TOE normalized the up-regulated mRNA for all examined genes except *Pik3ra* that was up-regulated. Mammary gland tissues of DMBA group showed excessive proliferation of lining epithelium of acini and ductules with hyperchromatic nuclei with excessive immunostaining of Bcl2 in the proliferated epithelium that was ameliorated by TOE administration. In conclusion, TOE regulated PI3K and Akt pathways involved in suppression of breast cancer growth and proliferation. TOE is effective as anticancer herbal agent.

## Introduction

Breast cancer is the most common form of malignancy and the leading cause of cancer-associated morbidity and mortality among women all over the world [1]. It attacks more than 500,000 women every year [2]. Breast cancer is characterized by excessive cell proliferation, dysregulation of cellular differentiation and insufficient apoptosis [3]. Experimentally induced mammary gland tumor in rodents has been used for several years to emulate human breast carcinogenesis. Mammary tumors can be induced in susceptible rat strains after single doses of carcinogens such as DMBA or nitrosomethylurea. Rat tumors are not extremely invasive, have short latency, rarely metastasize and are highly hormone-dependent [4]. The tumor induced by this model is morphologically and histologically similar to that observed in human estrogen-dependent breast cancer [4].

Dimethylbenz(α)anthracene (DMBA), a well-known polycyclic aromatic hydrocarbon, is a widespread genotoxic and tumorigenic environmental pollutant [5]. Mammary tumor induced by DMBA is an important preclinical animal model of breast cancer [6]. The resulting metabolite of DMBA induces DNA damage through adding adenine and guanine residues to DNA. The rat and human mammary gland tumors induced by DMBA express many biochemical and molecular markers, such as p53, BRCA, Bcl2 and p63 [5]. As known, carcinogenesis is impaired by apoptosis that results in malignancy [7].

Identification of oncogene and its associated possible pathways is critical for understanding therapy resistance and effective treatment. PI3K (phosphatidylinositol 3-kinase) is activated by the binding of a ligand or growth factor to its related receptor tyrosine kinases (RTKs), which include human epidermal growth factor receptor family, insulin and insulin-like growth factor 1 receptor [7]. PI3K phosphorylates phosphatidylinositol 4,5-bisphosphate (PIP2) to phosphatidylinositol 3,4,5-triphosphate (PIP3), which leads to phosphorylation of AKT (Protein Kinase B) [8]. PIP3 acts as a docking site for AKT, which is the basic signaling mediator of PI3K pathway and phosphoinositide-dependent kinase 1 (PDK1). Phosphorylation of AKT stimulates cell growth and protein synthesis by activating mTOR [9]. Therefore, the severity of cancer urged us to search for alternative supplement to cure cancer, because chemotherapy has various disadvantages. The usage of dietary regimen and efficient natural products is a powerful tool to reduce breast cancer mortality [10]. Recently, natural agents have received much attention because of their related antioxidant and anticancer properties [11]. Eighty percent of the world population partially uses herbs for treating diseases, so WHO recommends the use of scientifically evaluated medicinal plants in primary health care after evaluating quality, effectiveness and safety [12].

*Taraxacum officinale ssp. officinale* extract (TOE) is used worldwide as herbal remedy to treat medical problems [13]. TOE, a member of the *Asteraceae* family, is most common throughout the warm-temperate zones of the Northern Hemisphere, Asia and Europe [14].

Phenolic compounds have significant importance because they are responsible for scavenging free radicals and sequestering transition metal ion [15]. The phenolic compounds in TOE act as neuroprotective antioxidants or reducing agents [16]. Furthermore, other studies showed that TOE were reported to display antioxidative and anti-inflammatory activities [17].

Recent studies show an efficient anti-cancer activity of *T. officinale* root extract [18,19] but the exact mechanism is still unclear. Therefore, the present study aims to evaluate the genetic effects of *T. officinale* root extract on the PI3K/Akt pathway in DMBA-induced breast cancer in rats and also evaluating its biochemical, histopathological and immunohistochemical effects in this model of mammary carcinogenesis.

## Materials and methods

### Materials

The adult female rats were purchased from King Fahd Institute for Scientific Research, King Abdel Aziz University, Saudi Arabia. DMBA was purchased from Santa Cruz Biotechnology, Heidelberg, Germany. *T. officinale* roots were bought from Taif Markets and were identified by botanist (Prof, Yassin Asoudani, Taif University) and a specimen was added to herbarium of Turabah university college voucher # 543. Solvents and other related materials were from Sigma-Aldrich (St. Louis, MO, U.S.A.).

### Animals and experimental procedure

The present study has been approved by the Ethical Committee Office of the dean of scientific affairs of Taif University (project number 5523-438-1), Saudi Arabia. Eighty adult female Wistar rats weighing 150–200 g were kept under conditions of controlled temperature (25 ± 2°C) and relative humidity of 50 ± 10% with a 12-h/12-h day–night cycle in laboratory animal unit, College of Applied Medical Sciences, Turabah, Taif University. Animals have gained free access to tap water and standard laboratory chow (Teklad global diet 2,918, 18.6% protein, 44.2% carbohydrate, and 6.2% fat, 3.1 kcal/g, Envigo, U.K.). Animal studies were conducted according to the guidelines for the care and handling of animals prepared by the Animal Care Committee, Taif University.

### Preparation of *T. officinale ssp. officinale* root extract

*T. officinale* roots were thoroughly washed with distilled water. One hundred grams of roots were mixed in 200 ml of distilled water and homogenized using a blender. Resulted homogenate was filtered and spinning of the filtrate was done, 8000×***g*** for 5 min at 25°C. Filtering of the supernatant was done using 0.45 µm filters, followed by lyophilization. The dry powder was dissolved in water to get a stock solution of 100 mg/ml TOE [20].

### Experimental design

The present study was carried out on 80 adult healthy female albino rats, which were divided into four groups (*N* = 20). Negative control group maintained without treatment. *T. officinale* group; administered 500 mg/kg *T. officinale* root extract at the fourth month by oral gavage daily for 4 weeks. DMBA group (positive control group); administered single dose of DMBA (Sigma Chemical Co, St Louis, MO) in sesame oil (50 mg/kg b.wt) by oral gavage at 50 days of age. DMBA group treated with TOE; administered single dose of DMBA in sesame oil (50 mg/kg b.wt) by oral gavage at 50 days of age then treated daily with 500 mg/kg TOE by oral gavage after 4 months from DMBA administration and treatment continued for 4 weeks. Animals were checked weekly to detect tumors by palpation beginning 4 weeks after DMBA administration for confirmation of tumor incidence and beginning of treatment. Rats were killed 5 months post-administration of carcinogen, animals were killed after diethyl ether inhalation then blood and tissue specimens were collected. Tumor masses were weighed and stored for histopathological and molecular studies.

### Biochemical estimation of CA15-3

The concentrations of serum cancer antigen 15-3 (CA15-3; cobas e601, Roche, Switzerland) were detected by chemiluminescence method according to the manufacturer’s instructions.

### RNA extraction, cDNA synthesis and gene expression analysis

Total RNA was extracted from breast tissue samples (100 mg). Samples were flash frozen and stored in liquid nitrogen at −70°C in Qiazol till use. RNA was extracted based on our previous study [21]. Extracted RNA was checked for integrity using electrophoresis in denatured gel. RNA concentration was measured using Bio-Rad spectrophotometer with 260 nm. RNA samples with ratio of 1.60–1.90 were used for reverse transcription. For cDNA synthesis, 3 µg of total RNA and 0.5 ng of oligo dT primer were incubated in the PeX 0.5 thermal Cycler (PCR machine) at 70°C for 5 min for denaturation. Then, RT-buffer (4 µl), 10 mM dNTPs (2 µl) and Moloney Murine Leukemia Virus (M-MuLV, 100 U) were added and re-incubated in PCR machine at 37°C for 1 h, and at 90°C for 10 min to inactivate the enzyme. For semi-quantitative PCR analysis, specific primers stated on Table 1 were designed using Oligo-4 computer program (Macrogen Company, GAsa-dong, and Geumcheon-gu. South Korea). PCR reaction was conducted (cDNA; 1 µl, forward and reverse primer; 1 µl of 10 pM and PCR master mix; 12.5 µl was from Promega Corporation, Madison, WI) in a total volume 25 µl. The cycle sequence of PCR reaction was done by denaturation for 1 min at 94°C, annealing at the specific temperature (Table 1) and extension for 1 min at 72°C with additional final extension for 7 min at 72°C. As a reference, expression of glyceraldehyde-3-phosphate dehydrogenase (G3PDH) mRNA as housekeeping gene was detected. PCR products were visualized after electrophoresis in 1.5% agarose gel after staining with ethidium bromide in TBE buffer under UV light and photographed using gel documentation system.2.7 [21].

**Table 1:** Polymerase chain reaction conditions for the analyzed genes

Primer	Forward	Reverse	Annealing temperature	Band size
GAPDH	AGATCCACAACGGATACATT	TCCCTCAAGATTGTCAGCAA	52°C	309 bp
Pik3r1	CCCTCAGTGGACTTGGATGT	GCTGCTGGGAATCTGAAAAG	59°C	326 bp
Map3k1	AGTGCCAGCTCAGAGGACAT	GGCTTTGGCCTGTGTATGTT	59°C	407 bp
Erbb2	CCCATCAGAGTGATGTGTGG	TCATCTTCCAGCAGTGAACG	59°C	337 bp
PIk3ca	GAATTGGGAGAACCCAGACA	TGTCTTTCAGCCACTGATGC	58°C	308 bp
Pdk1	AAATGCGAAATCACCAGGAC	ATATGGGCAATCCGTAACCA	56°C	320 bp
Akt1	ACTCATTCCAGACCCACGAC	TGAGCTCGAACAGCTTCTCA	59°C	438 bp

### Histopathological examination

Mammary tissues were obtained from killed rats after killing using diethyl ether inhalation then fixed for 24 h in a 10% neutral buffered formalin solution. Subsequently tissues were routinely processed, washed, dehydrated in alcohol, cleared in xylene, paraffin embedded, casted and cut into 5 μm sections. The tissue sections were stained with hematoxylin and eosin (H and E). Tissue slides were visualized using a Leica DM1000 microscope, and photos were captured using AmScope MU1403 digital camera.

### Immunohistochemical examination of Bcl2

Mammary tissues specimens were fixed in 10% buffered neutral formalin, washed, dehydrated, cleared, embedded in paraffin, casted and finally sectioned. Deparaffinization was done using xylene. About 3% H_2_O_2_ was added for 10 min to inactivate the peroxidases. Then antigen retrieval was performed by heating at 121°C in 10 mM citrate buffer for 30 min then blocking was done for 20 min in 5% normal serum. After that sections were incubated with mouse monoclonal anti-Bcl2 primary antibody (sc-7382; Santa Cruz Biotechnology, Inc., Dallas, TX) in PBS overnight at 4°C. After washing with PBS, sections were incubated with a goat anti-rabbit IgG biotin-conjugated secondary antibody (1:2,000; sc 2040; Santa Cruz Biotechnology, Inc., Dallas, TX). After incubation with horseradish peroxidase-labeled streptavidin, antibody binding was visualized using diaminobenzidine, and sections were counterstained with hematoxylin [22].

### Statistical analysis

Results were shown as means ± standard error of means (SEM). Data analysis was done using SPSS software version 11.5 for Windows (SPSS, IBM, Chicago, IL, U.S.A.) using analysis of variance (ANOVA) and *post hoc* descriptive tests with *P*<0.05 considered as statistically significant. Regression analysis was calculated using the same software.

## Results

### The therapeutic effects of TOE on serum CA15-3 levels in experimentally induced breast cancer

Administration of *T. officinale* extract for 4 consecutive weeks decreased the elevated CA15-3 levels detected in DMBA administered rats. The carcinogenic group showed highly significant levels (*P*<0.01) of CA15-3 (34.6 ± 0.07 U/ml) compared with control rats and *T. officinale* extract group only (15.3 ± 0.03 and 13.3 ± 0.05 U/ml, respectively). Administration of *T. officinale* extract in breast cancer rats decreased significantly CA15-3 levels (19.8 ± 0.04 U/ml) compared with breast cancer group (*P*<0.05)**.**

### The therapeutic effect of *T. officinale* extract on alteration in *Pik3r1* and *Map3k1* mRNA expression in experimentally induced breast cancer

**Figure 1:**
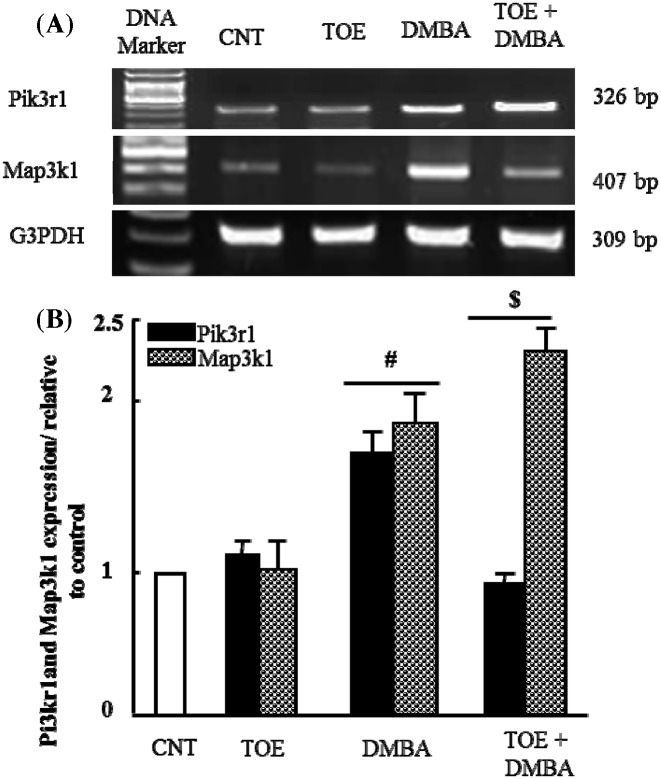
Results of gene expression analysis: *Pik3r1* and *Map3k1* (**A**) Semi-quantitative RT-PCR analysis of *Pik3r1* and *Map3k1* mRNA expressions and their corresponding *G3PDH* in mammary tissue of control, TOE, DMBA and DMBA treated with TOE groups. (**B**) Densitometric analysis was conducted for three different experiments, and data were presented as the mean ± standard error. ^#^*P*<0.05 vs. control group, and ^$^*P*< 0.05 vs. DMBA administered group.

It has been suggested that the PI3K/Akt pathway can be involved in tumor incidence. Therefore, we examined the expression of *Pik3r1* and *Map3k1* first**.** Figure 1A showed that administration of DMBA up-regulated mRNA expression of *Pik3r1* and *Map3k1* compared with control and TOE groups. It induced 1-fold increase in densitometric analysis. TOE administration for 4 weeks inhibited DMBA-altered *Map3k1* expression and normalized it significantly (*P*<0.05) as seen in Figure 1B. On the other hand, expression of *Pik3r1* was found to be increased in DMBA group treated with TOE compared with control, TOE and DMBA groups.

### The therapeutic effect of *T. officinale* extract on alteration in *Erbb2* and *PIK3ca* mRNA expression in experimentally induced breast cancer

**Figure 2:**
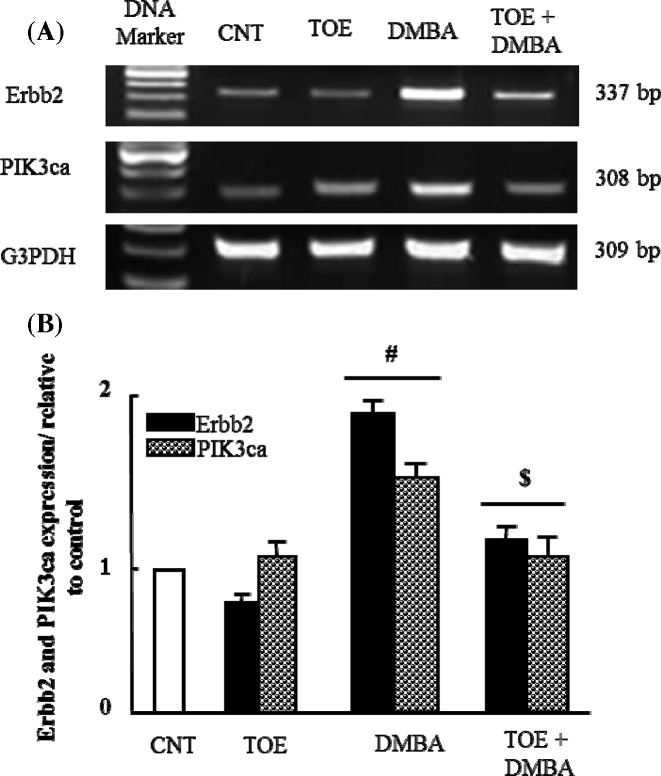
Results of gene expression analysis: *Erbb2* and *Pik3ca* (**A**) Semi-quantitative RT-PCR analysis of *Erbb2* and *Pik3ca* mRNA expressions and their corresponding *G3PDH* in mammary tissue of control, TOE, DMBA and DMBA treated with TOE groups. (**B**) Densitometric analysis was conducted for three different experiments, and data were presented as the mean ± standard error. ^#^*P*<0.05 vs. control group and ^$^*P*<0.05 vs. DMBA administered group.

Figure 2A showed that induction of mammary gland tumor by DMBA up-regulated significantly (*P*<0.05) mRNA expression of Erbb2 and PIK3ca. Four months after tumor induction, TOE was administered for 4 weeks and was found to normalize the expression of examined genes as seen in densitometric analysis (Figure 2B).

### The therapeutic effect of *T. officinale* extract on alteration in *Pdk1* and *Akt1* mRNA expression in experimentally induced breast cancer

**Figure 3:**
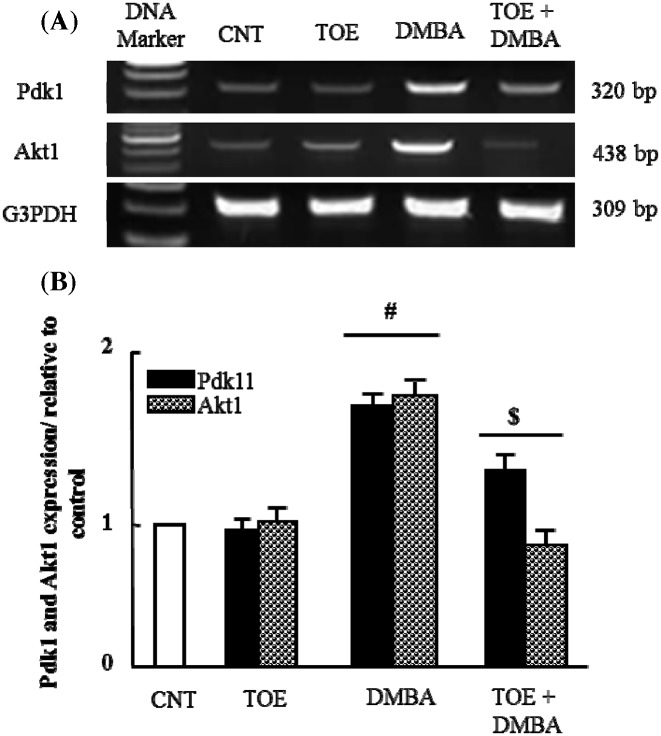
Results of gene expression analysis: *Pdk1* and *Akt1* (**A**) Semi-quantitative RT-PCR analysis of *Pdk1* and *Akt1* mRNA expressions and their corresponding *G3PDH* in mammary tissue of control, TOE, DMBA and DMBA treated with TOE groups. (**B**) Densitometric analysis was conducted for three different experiments, and data were presented as the mean ± standard error. ^#^*P*<0.05 vs. control group, and ^$^*P*<0.05 vs. DMBA administered group.

Finally, to confirm and complete the signaling pathway for tumor incidence, we examined the mRNA expression of *Pdk1* and *Akt1* after DMBA administration. *Pdk1* and *Akt1* mRNA were up-regulated after DMBA administration and normalized after TOE supplementation (Figure 3). All these findings confirmed the involvement of the PI3K/Akt pathway in the mammary gland tumor incidence and TOE has the potential to act as a promising anti-carcinogenic herbal medication.

### The effect of TOE administration on tumor weight

There were no visible mammary tumors in control group and TOE administered group whereas, average tumor weight was 18.3 ± 3.8 g in DMBA administered group. Treatment with TOE decreased tumor size to an average of 6.3 ± 1.5 g as shown in Table 2.

**Table 2:** Effect of TOE administration on tumor weight

Group	Control	Tarax	DMBA	DMBA = Tarax
Tumor weight (g)	0	0	18.3 ± 3.8	6.3 ± 1.5

### Histopathological findings

**Figure 4:**
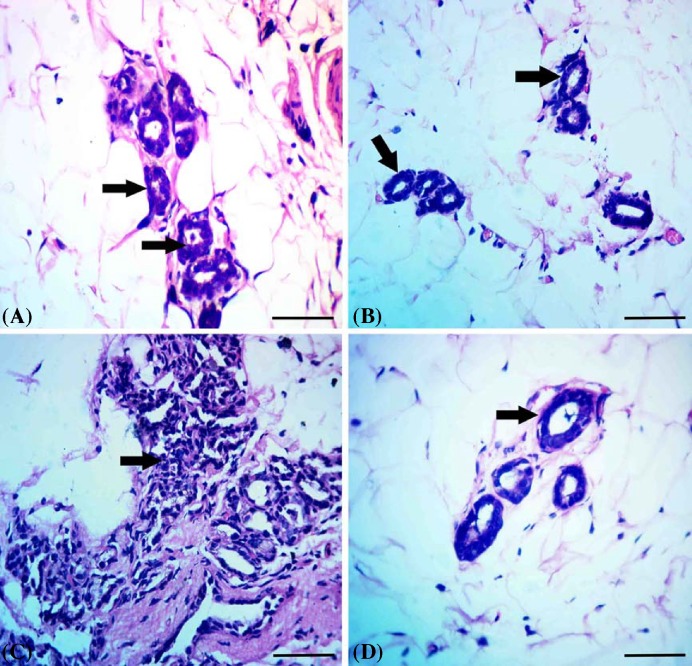
Results of histopathological examination (**A**) Mammary tissue of control group showing the normal picture of resting state with normal acini (arrows) and ductules. (**B**) Mammary tissue of *T. officinale* extract administered group showed normal tissue architecture with normal acini (arrows). (**C**) Mammary tissue of DMBA administered group showed excessive proliferation of lining epithelium of acini and ductules with hyperchromatic nuclei (arrow). (**D**) Mammary tissue of DMBA administered group treated with *T. officinale* extract showed restoration of normal tissue picture with normal acini (arrow); scale bar = 100 µm.

Mammary tissue of control group had the normal picture of resting state with normal acini and ductules (Figure 4A). Mammary tissue of TOE administered group showed normal tissue architecture with normal acini and ductules (Figure 4B). Mammary tissue of DMBA administered group showed excessive proliferation of lining epithelium of acini and ductules with hyperchromatic nuclei (Figure 4C). Mammary tissue of DMBA administered group treated with *T. officinale* extract showed restoration of normal tissue picture with normal acini and ductules (Figure 4D).

### Results of immunohistochemical examination of Bcl2

**Figure 5:**
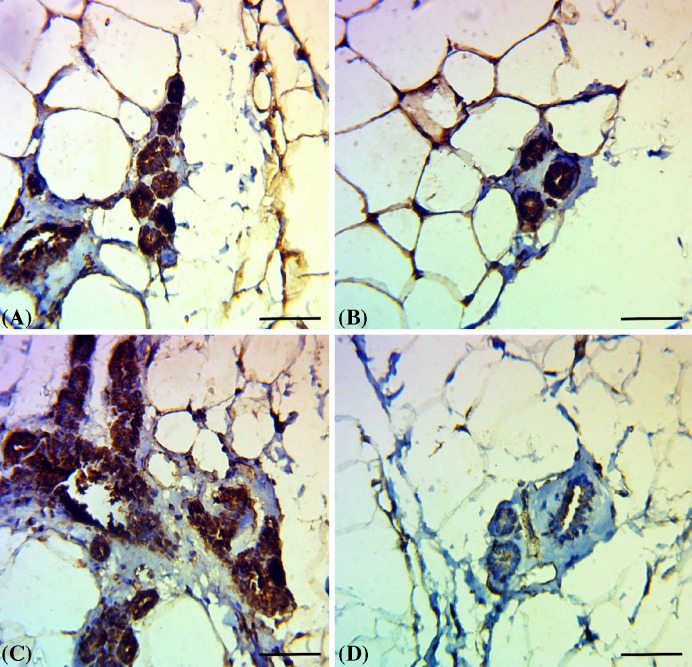
Immunohistochemical examination of Bcl2 expression (**A** and **B**) Mammary tissue of control group and *T. officinale* extract administered group showed increased expression of Bcl2 in both acinar and ductal epithelium. (**C**) Mammary tissue of DMBA administered group showed excessive immunostaining of Bcl2 in the proliferated epithelium of acini and ductules. (**D**) Mammary tissue of DMBA administered group treated with *T. officinale* extract showed weak expression of Bcl2 in acini and ductules; scale bar = 100 µm.

Mammary tissue of control and TOE administered groups showed increased expression of Bcl2 in both acinar and ductal epithelium (Figure 5A & 5B). Mammary tissue of DMBA administered group showed excessive immunostaining of Bcl2 in the proliferated epithelium of acini and ductules (Figure 5C). Mammary tissue of DMBA administered group treated with *T. officinale* extract showed weak expression of Bcl2 in acini and ductules (Figure 5D).

## Discussion

Breast cancer is widely common tumor among women, and one of the most leading causes of female cancer death [23]. According the American Cancer Society, breast cancer is still the most prevalent malignant neoplasm, representing ~29% of new carcinoma cases and has the most prevalent lethal cancer-related mortality in female worldwide [24].

Experimental tumor markers are frequently applied for screening and monitoring of many cancers and possible factor that may regulate it. In our study, administration of *T. officinale* extract decreased significantly CA15-3 levels (19.8 ± 0.04 U/ml) compared to breast cancer group (34.6 ± 0.07 U/ml). CA15-3 is a Food and Drug Administration (FDA)-approved tumor markers used for breast cancer monitoring [25]. CA15-3 is a mucinous glycoprotein produced by the Mucin1 gene (*MUC-1*). Mucin1 is mostly found in epithelial cells, and its expression is increased in breast cancer, colon, lung, pancreatic and ovarian cancers [26].

The PI3K/PTEN/AKT signaling pathway has several roles in different cellular activities, including survival, cytoskeleton rearrangement, cell proliferation, metabolism and membrane transit [27]. The abnormal activation of this pathway leads to many affections such as diabetes, autoimmune diseases and cancer. So, there is a big challenge to discover new gene biomarkers to prognosticate effective treatment to overcome drug resistance [28].

In the present study, we show that expression of *PIK3R1* increased in DMBA group treated with TOE in comparison with control, TOE and DMBA groups. The *PIK3R1* gene is known to play a tumor suppressor role because the PI3K subunit p85α (p85α) regulates and stabilizes p110α [29]. The products of *PIK3R1* act as a negative controller of PI3K activity, either by organizing the levels of PIP3, which mediates AKT phosphorylation, or by directly increasing activity of PI3K [30].

Our results showed that treatment with TOE for 4 weeks normalized significantly (*P*<0.05) the elevated Map3k1 expression caused by DMBA administration. The MAPK cascades are major signaling pathways that play essential cellular roles, including proliferation, differentiation, migration and apoptosis [8].

In our study, induction of mammary gland tumor by DMBA significantly up-regulated (*P*<0.05) mRNA expression of Erbb2 and *PIK3ca*. TOE was found to normalize the up-regulated expression of these genes. ERBB2 is one of the HER family of receptor tyrosine kinases, which is overexpressed in different tumors [31]. About 30% of breast cancer cases showed ERBB2 up-regulation that has become an important indication of chemoresistance and worse prognosis of breast cancer [32]. Abnormal activation of ERBB2 and PI3K/AKT cascade pathway is commonly related to tumorigenesis, drug resistance and carcinoma progression [33].

*PIK3CA* encodes for the 110 kDa p110α subunit of the class 1 phosphatidylinositol 3-kinase (PI3K), which is mainly involved in regulating molecular growth and survival signaling. The PI3K pathway expresses proliferative and migratory signals and is frequently activated in breast cancer [34].

Our findings showed that *Pdk1* and *Akt1* mRNA expression was up-regulated after DMBA administration and normalized after TOE supplementation. The protein kinase 3-phosphoinositide-dependent protein kinase-1 (PDK1) plays a fundamental role in signaling pathways activated by different growth factors and hormones. PDK1 acts together with PI3K and activates protein kinase B (AKT). Several studies showed that PDK1 is overexpressed in particular cancers and activates growth and survival of cancer cells independent of Akt signaling. These results provide evidence that PDK1 is not only an Akt-activating agent, but also an essential oncogenetic regulator and a potential therapeutic target in cancer. Akt1 is a member of the serine-threonine kinase class that acts as a key regulator of many cellular activities, including growth, proliferation, survival and angiogenesis [35]. AKT has a significant role in glucose metabolism, cell proliferation, survival and programmed cell death [36]. Active form of AKT is the phosphorylated form which frequently occurs in several types of cancer cells [36]. AKT1 activation accelerates tumorigenesis and act as an apoptosis inhibitor. Activation of AKT can also occur via constructive activation of PI3K through activation and mutations of receptor tyrosine kinase predominantly in the *PIK3CA* gene [37].

The use of plant extracts changed the genetic pathways associated with cancer evidence and resistance such as apoptosis. Apoptosis is the programmed cell death that is activated and/or suppressed by different proteins as caspase cascade pathway. It regulates caspase pathways by stimulation or inhibition of different apoptotic genes such as Bcl2, P53, AKT1 or BID [38]. Induction of apoptosis in cancer is a main target for suppression of tumor progression [38].

Mammary tissue of DMBA-administered rats showed excessive proliferation of lining epithelium of acini and ductules with hyperchromatic nuclei with excessive immunostaining of Bcl2 in the proliferated epithelium that was ameliorated by TOE administration. Bcl2 is the leading member of Bcl2 apoptosis regulating protein family that regulates programmed cell death, either by inducing or inhibiting apoptotic cell death [39]. Bcl2 is a major anti-apoptotic protein located at position 18q21.33 that encodes the Bcl2 protein, which is an integral outer mitochondrial membrane protein that prevents programmed death of different cells including cancer cells and inhibits the release of cytochrome *C*. The expression of the Bcl-2 proteins are mainly associated with incidence and progression of breast cancer [40]. Other studies showed suppressed viability of gastric cancer cells when treated with TOE [41] and apoptosis inducing effects of TOE in some types of cancer such as colorectal cancer [18].

## Conclusion

In conclusion, *T. officinale* extract has the potential to inhibit mitogen-activated protein kinases and phosphatidylinositol-4, 5-bisphosphate 3-kinase/protein kinase B pathways, leading to the suppression of cell growth and proliferation. *T. officinale* extract is recommended as a potential herbal medication that needs further evaluation for use in human breast cancer cases.

## Abbreviations

DMBA: dimethylbenz (α) anthracene
PDK1: phosphoinositide-dependent protein kinase-1
RTK: receptor tyrosine kinase
TMBA: dimethylbenz (α) anthracene
TOE: *Taraxacum officinale ssp. officinale* root extract
